# *APOE* genotype and stress response - a mini review

**DOI:** 10.1186/s12944-016-0288-2

**Published:** 2016-07-25

**Authors:** Janina Dose, Patricia Huebbe, Almut Nebel, Gerald Rimbach

**Affiliations:** 1 Institute of Human Nutrition and Food Science, Kiel University, Hermann-Rodewald-Str. 6, D-24118 Kiel, Germany; 2 Institute of Clinical Molecular Biology, Kiel University, Schittenhelmstr. 12, D-24105 Kiel, Germany

**Keywords:** Apolipoprotein E isoform, Oxidative stress, Endoplasmic reticulum stress, Mitochondrial function, Immune function, Therapeutic intervention

## Abstract

The *APOE* gene is one of currently only two genes that have consistently been associated with longevity. Apolipoprotein E (APOE) is a plasma protein which plays an important role in lipid and lipoprotein metabolism. In humans, there are three major APOE isoforms, designated APOE2, APOE3, and APOE4. Of these three isoforms, APOE3 is most common while APOE4 was shown to be associated with age-related diseases, including cardiovascular and Alzheimer’s disease, and therefore an increased mortality risk with advanced age. Evidence accumulates, showing that oxidative stress and, correspondingly, mitochondrial function is affected in an APOE isoform-dependent manner. Accordingly, several stress response pathways implicated in the aging process, including the endoplasmic reticulum stress response and immune function, appear to be influenced by the *APOE* genotype. The investigation and development of treatment strategies targeting APOE4 have not resolved any therapeutic yet that could be entirely recommended. This mini-review provides an overview on the state of research concerning the impact of the *APOE* genotype on stress response-related processes, emphasizing the strong interconnection between mitochondrial function, endoplasmic reticulum stress and the immune response. Furthermore, this review addresses potential treatment strategies and associated pitfalls as well as lifestyle interventions that could benefit people with an at risk APOE4 genotype.

## Background

### General relevance of the topic

Aging is characterized by both a proceeding decline in biological functions and a decreased stress resistance [1]. This raises the organisms’ susceptibility to disease and is reflected in an increase in all-cause mortality with advanced age [2]. Accordingly, aging is defined as the “accumulation of diverse deleterious changes in cells and tissues with advancing age that increase the risk of disease and death” [3]. Increasing evidence suggests a certain degree of heritability of lifespan [4–6]. Genetic difference is assumed to account for approximately 15–25 % of the variance in human lifespan [7, 8]. So far, candidate gene (CGAS) and genome-wide association studies (GWAS) have identified variation in only two genes, namely forkhead box O3 (*FOXO3*) and apolipoprotein E (*APOE*) to be consistently associated with human longevity [8–14]. Of these two *loci*, *APOE* was the first to be identified [9]. Not surprisingly, *APOE* has since then been extensively studied in the context of aging [15].

### Role of APOE in lipid metabolism

**Fig. 1:**
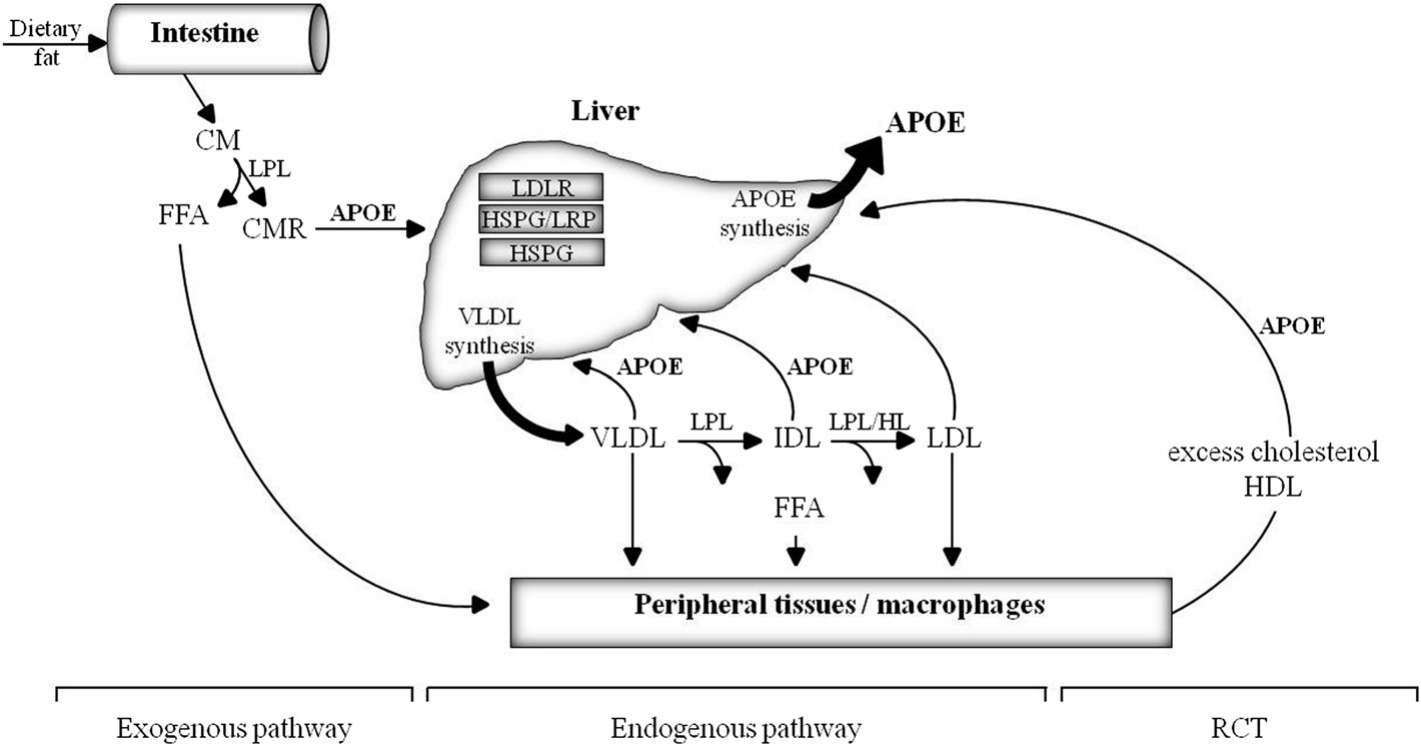
Overview on the role of apolipoprotein E (APOE) in the three main pathways of plasma lipoprotein metabolism. In the exogenous pathway, chylomicrons (CM) are generated in the intestine from dietary fat and cholesterol and enter the systemic circulation, where they acquire APOE. CM are lipolyzed by lipoprotein lipase (LPL) and form CM remnants (CMR). Peripheral tissues, e.g., skeletal muscle and adipose tissue, take up released free fatty acids (FFA) and cholesterol. CMR undergo hepatic clearance after APOE-mediated binding to cell surface receptors, e.g., low density lipoprotein (LDL) receptor (LDLR) or LDLR-related protein (LRP) and heparan sulfate proteoglycan (HSPG) pathways. In the endogenous pathway, very low density lipoproteins (VLDL) are synthesized and secreted by the liver. LPL and hepatic lipase (HL) cause the release of FFA and the formation of VLDL remnants which can be cleared by the liver by APOE-mediated uptake (see above). Complete hydrolysis of VLDL results in the formation of LDL which lack APOE (LDL contain APOB-100 which mediates cellular uptake). The reverse cholesterol transport (RCT) enables excess cholesterol to be redirected from peripheral tissues to the liver via high density lipoproteins (HDL) that comprise APOE. *APOE* apolipoprotein E, *CM* chylomicron, *CMR* CM remnant, *FFA* free fatty acids, *HDL* high density lipoprotein, *HL* hepatic lipase, *HSPG* heparan sulfate proteoglycan, *IDL* intermediate density lipoprotein, *LDL* low density lipoprotein, *LDLR* LDL receptor, *LPL* lipoprotein lipase, *LRP* LDL receptor-related protein, *RCT* reverse cholesterol transport. Figure prepared according to [22, 23, 176]

In the 1970s, APOE was discovered as a constituent of lipoproteins and potent modulator of plasma lipoprotein and cholesterol concentrations. Up to 75 % of the plasma APOE is synthesized by liver parenchymal cells [16]; however, other organs and tissues produce significant amounts of APOE, most notably the brain, but also spleen, kidneys, macrophages and adipocytes [17–19]. In its primary role as an apolipoprotein, APOE maintains the structural integrity of lipoproteins and facilitates their solubilization in the blood [20]. APOE is fundamentally involved in the lipid homeostasis of hepatic and non-hepatic tissues. Both the exogenous and the endogenous pathway of lipoprotein metabolism depend on APOE. Chylomicrons, synthesized and secreted by the intestine to transport dietary lipids to the liver and adipose tissues, acquire APOE in the circulation. Very low density lipoprotein (VLDL) particles secreted by the liver comprise APOE and transport endogenously synthesized triglycerides, phospholipids, and cholesterol and cholesteryl esters to peripheral tissues [19]. As a high-affinity ligand for receptors of the low density lipoprotein (LDL) receptor family, APOE facilitates the internalization of lipids into hepatic and extrahepatic cells [21]. Moreover, APOE produced by macrophages plays a pivotal role in the so-called reverse cholesterol transport, where excess cholesterol from peripheral tissues is redirected via APOE-containing high density lipoprotein (HDL) particles to the liver for elimination [22, 23]. By these functions, APOE is fundamentally involved in the lipid and cholesterol homeostasis. Figure 1 provides the interested reader with a more extensive overview on the role of APOE in plasma lipoprotein metabolism.

### Additional functions of APOE

Over the course of time, studies on *Apoe* knockout (KO) mice and in vitro studies revealed functions of APOE which can not entirely be attributed to its role in lipid metabolism and strongly suggest APOE to be involved in the aging process. *Apoe* KO mice lack endogenous *Apoe* expression due to gene targeting-induced inactivation of the murine *Apoe* gene [24]. These mice display age-related phenotypes, including an earlier loss of hair follicles, graying, arrested spermatogenesis [19] and also a shortened lifespan [19, 25]. *Apoe* KO mice show an altered lipoprotein profile compared with wild type mice and spontaneously develop atherosclerosis [26], which made them a powerful tool in cardiovascular disease (CVD) research [20]. Moreover, *Apoe* KO mice suffer from neurological disorders [27]; they develop type II diabetes [25] and show defects in the immune response, leaving them more prone to bacterial infections [28, 29] and LPS-induced death [30]. Furthermore, *Apoe* KO mice showed elevated markers of oxidative stress [31] and in vitro studies later substantiated APOE to bear anti-oxidative properties [32]. Although the impact of oxidative stress and damage on lifespan remains controversial, its influence on the development of certain (age-related) pathophysiological conditions is undisputed [33]. Oxidative stress has been shown to augment APOE secretion from adipocytes and APOE overexpression protected cells from hydrogen peroxide-induced damage [34]. By its pleiotropic nature, the *APOE* gene affects multiple phenotypic traits simultaneously depending on the site of APOE protein synthesis. The multiple functions of the APOE protein make it a potent modulator of cellular stress response processes and the aging process.

### APOE structure and polymorphism

The human *APOE* gene is located on the long arm of chromosome 19, in close proximity to the genes of apolipoprotein C-I and C-II. The low density lipoprotein receptor (LDLR) is located on the short arm of the same chromosome, suggesting chromosome 19 to play an important role in lipoprotein metabolism [35]. The *APOE* gene is composed of four exons, separated by three introns [36]. The mature APOE protein found in plasma is 299 amino acids long (Molecular weight ~34000 Dalton). However, the primary translated *APOE* gene product comprises 317 amino acids with an N-terminal 18 amino acids long signaling peptide being removed co-translationally. Prior to secretion, the APOE protein undergoes *O*-linked glycosylation and sialylation in the Golgi apparatus; but most plasma APOE is post-secretory de-sialylated [37]. The exon 4 of the *APOE* gene also inherits the two non-synonymous single nucleotide polymorphisms, which gave rise to the three major *APOE* alleles *ε2*, *ε3,* and *ε4* (rs429358C>T: distinguishes *ε3* and *ε4*, rs7412C>T: distinguishes *ε3* and *ε2*). These alleles encode six major *APOE* genotypes (three homozygous and three heterozygous). Basis of the APOE protein isoforms are single amino acid interchanges at positions 112 and 158 (APOE3 has cysteine on residue 112 and arginine at residue 158, while APOE2 carries cysteine and APOE4 arginine on both positions; [38, 39]). Worldwide, allelic variation in the *APOE* locus ranges from 0 to 20 % for *ε2*, 60-90 % for *ε3,* and 10–20 % for *ε4* alleles, respectively [40].

The amino acid interchanges influence both the structure and the function of the resulting isoproteins. Lipid-free APOE consists of two independently folded domains, which are separated by a hinge region. The N-terminal LDL-receptor binding domain is arranged in a four-helix bundle. The major C-terminal domain contains amphipathic α-helices, which mediate the high-affinity binding of APOE to lipoproteins [39, 41]. APOE isoforms show different lipoprotein binding preferences. While APOE3 and APOE2 preferentially bind high density lipoprotein (HDL), APOE4 rather associates with VLDL particles [42]. The enhanced association of APOE4 with VLDL is likely a consequence of the greater lipid-binding ability based on the protein structure, with the stability of the APOE protein or its readiness to unfold appearing to be the determining factor [43]. It has been shown that the molecular stability of the APOE isoproteins decreases in the order APOE2>APOE3>APOE4 [43]. Next to a decreased stability, APOE4 tends to form a molten globule state [44]. It had been suggested that in APOE4 a unique interaction occurs between the N- and C-terminal domain which is not as pronounced in APOE3 and APOE2. More precisely, a salt bridge formation between the amino acid residues Arg61 (N-terminal domain) and Glu255 (C-terminal domain) (for a visual depiction of these proposed structures of APOE3 and APOE4 see [39]) was suggested to be responsible for the direction of the lipoprotein preference of APOE4 towards VLDL. This interaction was referred to as APOE4 domain interaction [41, 42, 45].

Later, the NMR structure of full-length APOE3 revealed several interactions between the two domains to also occur in APOE3 [41, 46]. Mizuguchi et al. [47] reported a greater stabilizing effect of the C-terminal domain on the N-terminal domain in APOE3 compared with APOE4. Instead of a direct interaction of the C- and N-terminal domain in APOE4, Frieden and Garai [41] suggested that the positive charge of arginine at position 112 in the APOE4 protein is propagated to the C-terminal domain resulting in structural changes. This view is substantiated by recent computational simulations which indeed showed more contacts between the N- and C- terminal domain in APOE4, but with Arg61 and Glu255 appearing rather noninvolved [48]. Overall, both the N-terminal and C-terminal domain appear to be less stable in APOE4 and the reduced stability of the two domains is likely influencing its lipoprotein-binding abilities [49]. The APOE isoforms also differ in their LDL receptor binding affinities. While APOE3 and APOE4 bind to the LDL receptor with similar affinity, APOE2 appears defective in its receptor binding ability, displaying only about 1 % of the binding ability of APOE3 and APOE4 [50]. Both the specific lipoprotein binding preferences and the differential LDL receptor binding affinities contribute to the modulation of plasma lipid levels in response to variation in the *APOE* gene. Interestingly, APOE isoforms confer different disease susceptibilities. APOE2 is associated with increased plasma levels of cholesterol and triglycerides, which makes it a risk factor for type III hyperlipoproteinemia [50]. APOE4 is associated with an increased risk for cardiovascular disease (CVD), generally attributed to the higher plasma triglyceride and LDL cholesterol concentrations observed in *APOE ε4* carriers [20]. More obvious, the *ε4* allele increases the risk and decreases the age of onset of late-onset Alzheimer’s disease (AD) in a dose-dependent manner [51]. Not unexpectedly, the frequency of *APOE ε4* carriers decreases with age [52]. Therefore, Gerdes et al. [53] suggest entitling *APOE* a “frailty gene” rather than referring to it as a determinant of longevity.

## Main text

### *APOE* genotype and cellular stress response - linking *APOE* to the aging process

Increasing evidence links the *APOE* genotype to the cellular stress response and to the aging process. Overall, APOE4 appears rather defective in its response to stressors as it will be discussed below.

### *APOE* genotype and response to oxidative stress

Shortly after studies on *Apoe* KO mice had revealed antioxidative properties of APOE [31], in vitro studies indicated isoform-specific antioxidative properties in the order APOE2>APOE3>APOE4 [32]. Later, this was attributed to the number of free –SH groups available in the respective APOE isoproteins [54]. Since then, a huge number of studies has demonstrated an association between the *APOE* genotype and oxidative stress (reviewed in [55]). Consistent with being a poorer antioxidant compared with APOE3 and APOE2, APOE4 was also less effective in protecting cells from oxidative toxicity and death both in vitro [32, 56] and in vivo [57]. Accordingly, oxidative stress was elevated in APOE4 vs. APOE3 expressing macrophages [58]. Compared with the relative unambiguous in vitro situation, in vivo data concerning the association of the *APOE* genotype with oxidative stress appears rather inconsistent. While some reports show that APOE4 is positively associated with markers of oxidative stress (elevated levels of lipid peroxides in *APOE ε4*/*ε3* heterozygous individuals; [59]), other studies report no significant differences between the two APOE isoforms (similar levels of F_2_-isoprostane and thiobarbituric acid-reactive substances in APOE3 and APOE4 targeted replacement (TR) mice; [60]). It appeared that detrimental effects of APOE4, at least concerning CVD risk, may depend on the co-occurrence of additional stimuli [55], like tobacco smoke [61, 62] or elevated plasma cholesterol [63]. However, according to a recent meta-analysis on the association of the *APOE* genotype and coronary heart disease (CHD) risk, there is no clear evidence of smoking being in fact a modifier of the *APOE* genotype/CHD risk-association [64]. Studies on the influence of the *APOE* genotype on antioxidant enzyme activities in APOE TR mice and brains of AD patients yielded conflicting results [57, 58, 65]. Nrf2-dependent gene expression, however, appeared to be diminished in presence of APOE4 [66], and likewise levels of the anti-oxidative and anti-inflammatory metallothioneins were lower in various tissues of APOE4 vs. APOE3 TR mice [67]. Interestingly, APOE has also been shown to induce serum paraoxonase 1 (PON1) activity [68], and lower PON1 levels were observed in presence of APOE4 [69]. However, whether this difference in PON1 levels is reflected in a differential interaction of the APOE isoforms with PON1 remains ambiguous. One study reported PON1 to bind to both APOE3- and APOE4-HDL with similar activity and both complexes inhibited LDL oxidation to a similar extent [68].

### Association of the *APOE* genotype with the ER stress response and mitochondrial function

Besides the differential association of the APOE isoforms with oxidative stress and the potential *APOE* genotype-dependent regulation of antioxidant defense mechanisms, increasing evidence suggests the *APOE* genotype to influence mitochondrial and endoplasmic reticulum (ER) stress-related processes in an APOE isoform-specific manner. Mitochondrial dysfunction and disturbances in the ubiquitin proteasome system which both may evoke by oxidative stress are considered hallmarks of the aging process. Both systems have been implicated in the pathogenesis of various age-related diseases, particularly neurodegenerative disorders, such as AD and Parkinson’s disease [70]. The ER and mitochondria are interconnected both structurally and functionally [71]. Structurally, they interact via mitochondria-associated ER membranes (MAM), with mitofusin 2 (MFN2) functioning as a direct anchoring protein. One important way of communication between the two organelles is Ca^2+^ exchange via the calcium transfer channels voltage-dependent anion channel 1 (VDAC1) and inositol 1,4,5-triphosphate receptor Ca^2+^ channel (IP3R), located on both the mitochondrial and ER site of the MAM [71, 72]. Therefore, ER dysfunction may easily pass on to mitochondria and *vice versa*. The two organelles both significantly contribute to the endogenous production of reactive oxygen species (ROS) [73].

The ER mediates the synthesis, posttranslational modification and folding of almost all secretory and membrane proteins and is in these processes highly dependent on intra-compartmental calcium levels, an oxidative environment and the availability of specialized proteins, so-called chaperones, which facilitate proper protein folding. Changes in ER calcium levels, redox state but also metabolic or inflammatory changes may either lead to compromised ER function or an increased demand for (folded) proteins. Exceedance of the ER folding capacity causes an accumulation of unfolded or misfolded proteins in the ER lumen, referred to as ER stress [73]. In turn, the cell activates the so-called unfolded protein response (UPR) to restore homeostasis by inhibiting translation in general but simultaneously activating UPR target gene transcription. The UPR is based on ER stress sensing by three ER transmembrane proteins, serine/threonine-protein kinase/endoribonuclease (IRE1ɑ), eukaryotic translation initiation factor 2-alpha kinase 3 (PERK), and activating transcription factor 6 (ATF6), which each activates specific downstream signaling cascades when released from the chaperone 78 kDa glucose-regulated protein (GRP78; functioning as a luminal inhibitor). IRE1ɑ activates splicing of various mRNAs in the cytoplasm, including the mRNA of X-box binding protein 1 (XBP1), which is thereby activated to XBP1(S). PERK, by inhibition of the translation initiating factor eukaryotic translation initiation factor 2A (EIF2A), promotes general translation inhibition while favoring activating transcription factor 4 (ATF4) transcription. ATF6 is cleaved by proteolysis upon activation. The cytoplasmic fraction of ATF6 (ATF6 p50), XBP1(S), and ATF4 translocate into the nucleus and induce transcriptional activation of UPR target genes, including GRP78. Prolonged activation of the UPR may lead to cell death by transcriptional induction of the pro-apoptotic factor DNA damage-inducible transcript 3 protein (DDIT3) [71]. Interestingly, all three pathways of the UPR can trigger inflammatory processes by activating NF-kB [74].

It has been hypothesized that APOE4, due to its structural characteristics, might be recognized by the ER as misfolded, subsequently leading to the activation of the ER stress response [75]. Furthermore, the different folding status of APOE4 was shown to affect its trafficking through the secretory pathway [76]. Table 1 provides an overview of studies with relevant findings concerning the association of the *APOE* genotype with ER stress. Interestingly, there is evidence to suggest that ER stress is indeed an early feature of APOE4 pathogenicity. APOE4 compared with APOE3 TR mice showed enhanced phosphorylation of EIF2A in the brain as early as 4 months of age and already displayed mild cognitive impairment [77]. Moreover, Zhong et al. [75] reported all three pathways of the UPR up-regulated in primary Arg61 APOE mouse astrocytes. In these mice the threonine at position 61, present in the APOE amino acid sequence of all mammals despite humans and rabbits (which carry arginine at this position), is replaced by arginine. In consequence, these mice display APOE4 domain interaction [75], which was hypothesized to be a characteristic feature of the APOE4 protein [42, 45], as it has been already stated earlier. Furthermore, intracellular trafficking of APOE4 has been shown to be impaired in transfected murine Neuro-2a cells and primary neurons of APOE transgenic mice. However, this appeared not to be accompanied by elevated ER stress. Interestingly, normal trafficking of APOE4 was restored by either treating APOE4 with small-molecule structure correctors, which shall convey the structure of APOE4 into an APOE3-like state or by the introduction of threonine at position 61 [76]. Data on the association of the *APOE* genotype with ER stress in tissues other than the brain is rather limited. Two studies on the influence of the *APOE* genotype on ER stress parameters in peritoneal macrophages report conflicting results. Eberlé et al. [78] found ER stress unaffected by APOE4 domain interaction in resident peritoneal macrophages of Arg61 APOE mice, though cellular dysfunction in terms of reduced APOE secretion was observed. However, hypomorphic mutant mice used in the study secrete low amounts of APOE which might have impacted the results [79]. By contrast, peritoneal macrophages of APOE4 TR mice showed reduced phagocytic clearance of dead cells and elevated apoptosis sensitivity. These effects were ameliorated by treatment of the cells with the ER chaperone tauroursodeoxycholic acid [79]. A reduced trafficking velocity of APOE4 has also been observed in transfected human liver cells [80]. Human astrocyte transcriptome data substantiated an *APOE* genotype-dependent differential expression of markers of ER stress (e.g., ATF4) [81].

**Table 1:** Selected studies on APOE and endoplasmic reticulum stress

Reference	Model system	ER stress-relevant findings in E4-carrying cells
Sabaretnam et al. [80]	APOE4 vs. APOE3 transfected HepG2 cells	- Trafficking velocity ↓
Zhong et al. [75]	Primary astrocytes from Arg-61 APOE mice vs. wild type mice astrocytes	- Intracellular APOE concentration↓ - Levels and fragmentation of Oasis ↑ - Levels of UPR components and downstream effectors (ATF4, XBP1, BIP, GRP94, PDI, GADD34, Herp, CHOP) ↑
Brodbeck et al. [76]	EGFP-APOE4 vs. EGFP-APOE3 transfected Neuro-2a cells and primary murine hippocampal neurons	- Retention of APOE in Golgi and ER ↑ - Trafficking of APOE in the Golgi/Soma ↓ - PERK, ATF6, XBP1, IRE1ɑ, CHOP, BIP protein levels ↔ Impaired EGFP-APOE4 trafficking rescued by R61T mutation or APOE4 structure correctors.
Eberlé et al. [78]	Primary peritoneal macrophages from Arg-61 APOE vs. Thr-61 APOE mice	- APOE secretion ↓ - ATF4, CHOP, TRB3 mRNA levels ↔
Cash et al. [79]	Primary peritoneal macrophages from APOE4 vs. APOE3 TR mice	- Inflammation-induced apoptosis ↑ - Efferocytosis ↓ - Basal and stimulated conditions: JNK phosphorylation ↑ - Stimulated conditions: IRE1ɑ, PERK phosphorylation ↑ XBP1(S), CHOP mRNA and protein levels ↑ Improved efferocytosis, reduced apoptosis after tauroursodeoxycholic acid treatment.
Simpson et al. [81]	Human astrocyte transcriptome (lateral temporal cortex; *APOE ε4* carriers vs. non-carriers)	Altered pathways incl. transcription (ATF4, FOXN3) and ubiquitin-mediated proteolysis (ATG7, UBA5)
Segev et al. [77]	APOE4 vs. APOE3 TR mice (age: 4 months, cortex and hippocampus)	- Phosphorylation of eIF2ɑ, GCN2, PKR ↑

*ATF4* activating transcription factor 4, *ATF6* activating transcription factor 6, *ATG7* autophagy related 7, *APOE* apolipoprotein E, *BIP* binding immunoglobulin protein, *CHOP* c/EBP-homologous protein 10, *EGFP* enhanced green fluorescent protein, *ER* endoplasmic reticulum, *FOXN3* forkhead box N3, *GADD34* protein phosphatase 1, regulatory subunit 15A, *GCN* eukaryotic translation initiation factor 2 alpha kinase 4, *GRP94* heat shock protein 90, beta, *HERP* homocysteine-inducible, endoplasmic reticulum stress-inducible, ubiquitin-like domain member 1, *IRE1α* serine/threonine-protein kinase/endoribonuclease, *JNK* mitogen-activated protein kinase 8, *OASIS* old astrocyte specifically induced substance, *PDI* protein disulfide isomerase associated 3, *PERK* eukaryotic translation initiation factor 2-alpha kinase 3, *PKR* eukaryotic translation initiation factor 2-alpha kinase 2, *TRB3* tribbles homolog 3, *UBA5* ubiquitin like modifier activating enzyme 5, *XBP1* X-box binding protein 1

One way through which protein homeostasis may be impaired is oxidative damage which has been shown to be often tightly linked to mitochondrial dysfunction [70]. The primary role of mitochondria is energy production in form of adenosine triphosphate (ATP) via oxidative phosphorylation (OXPHOS). During this process, ROS are continuously generated as by-products. Additional functions of mitochondria include the regulation of metabolite levels, metal metabolism and Ca^2+^ homeostasis, processes that depend on proper OXPHOS function [82].

In situations of increased ATP demands, mitochondrial mass is increased by mitochondrial biogenesis. The key players in this process are peroxisome proliferator-activated receptors (PPARs), PPAR co-activators (e.g., PGC-1ɑ) and nuclear respiratory factors (NRFs), which are induced by the energy sensors adenosine monophosphate (AMP)-activated protein kinase (AMPK) and sirtuin 1 (SIRT1). Architecture and function of mitochondria are tightly linked and depend on the coordinated actions of fission- (including dynamin 1-like protein 1, DNM1L or DRP1; fission 1, mitochondrial, FIS1) and fusion- (including mitofusins, MFN1/2; optic atrophy 1 protein, OPA1) promoting proteins. Fission and fusion enable exchange of proteins and mitochondrial DNA between mitochondria and the production of proper sized organelles [82, 83]. Mitochondria are also involved in apoptosis and thereby in determining cell fate under stress conditions. Ca^2+^ overload and/or oxidative stress initiate steps of the apoptotic cascade, including cytochrome c release by mitochondria. This is usually based on the coordinated action of the pro- and anti-apoptotic proteins of the B cell lymphoma 2 family members (BCL2), opening of the mitochondrial permeability transition pore and organelle fragmentation [84, 85]. Damaged mitochondria are degraded through a mitochondria-specific autophagy-related pathway referred to as mitophagy. Importantly, evidence supports the hypothesis that defects in mitochondrial function, including OXPHOS deficiency, in one tissue may be signaled to the whole organism by mechanisms that are not yet entirely understood [82].

Due to the strong association of *APOE ε4* with AD, much of the data concerning mitochondrial function in relation to the *APOE* genotype was obtained in models of AD. Mitochondrial dysfunction has been reported to occur early in the pathogenesis of AD [86] and also appears to be an early sign of APOE4 pathogenicity [87]. Positron-emission tomography (PET) studies revealed abnormally low glucose metabolism in brains of *APOE ε4* carriers with [88–90] and without [91–95] diagnosed AD, detectable already at relatively young ages (<40 years) [96]. In the brain, glucose is the primary substrate for OXPHOS; thus, the brain depends on proper glucose metabolism [97]. Cell culture experiments revealed specifically APOE4 synthesized by neurons as being more prone to proteolytic cleavage compared with APOE3 [98–100]. Interestingly, zinc potently induced APOE4 proteolytic degradation in vitro [101] and, correspondingly, higher levels of zinc were detected previously in the serum of AD patients carrying the *ε4* allele [101, 102]. Specifically, APOE4 proteolytic fragmentation led to increased generation of neurotoxic C-terminally truncated fragments, particularly the fragment APOE (1-272) [98, 99]. Consistent with this finding, overexpression of APOE affected mitochondrial function in neurons in an isoform-dependent manner (mitochondrial dysfunction APOE4>APOE3) [99, 103]. The APOE4 (1-272) fragment appears to directly interact with mitochondria through binding to components of mitochondrial complexes III and IV, which resulted in decreased activities of the two respiratory complexes [103, 104]. More recently, decreased expression levels of respiratory complexes and a lowered respiratory capacity were also detected in murine neurons expressing full-length APOE4 (1-299) [87]. In the same study, astrocytes did not show any *APOE* genotype-specific effects, substantiating detrimental effects of APOE4 to be rather neuron-specific [87]. Down-regulated mitochondrial respiratory complexes in response to APOE4 have also been observed in gene expression profiling data of *post mortem* human hippocampus [105]. James et al. [106] performed a proteomic analysis of hippocampus-derived mitochondria of APOE3 and APOE4 transgenic mice under basal conditions and in response to an ischemic challenge. They found the proteomic signature of mitochondria between APOE3 and APOE4 mice to be already different under basal conditions and indicative for a differential regulation of energy production, metabolism and oxidative stress [106]. More recently, similar results were reported for synaptosomes of APOE transgenic mice [107]. Pathways of energy production, particularly of oxidative phosphorylation and ATP synthesis, were down-regulated in synaptic mitochondria of APOE4 vs. APOE3 transgenic mice [107]. In contrast, Liraz et al. [108] observed higher protein expression levels of the translocase of the outer mitochondrial membrane (Tomm40) and cytochrome c oxidase I (complex IV; mt-Co1 or CIV, resp.) in young APOE4 vs. APOE3 TR mice; however, this may represent a compensatory response to the stress induced by APOE4, as the authors discuss [108]. Consistent with an impaired respiratory function in presence of APOE4, we observed lower ATP levels in the brain of APOE4 vs. APOE3 TR mice in our previous studies [109]. Interestingly, a 3-months dietary curcumin supplementation induced mitochondrial biogenesis and expression of mitochondrial respiratory complexes only in APOE3 but not in APOE4 TR mice, suggesting a certain unresponsiveness of *APOE ε4* to improvements of mitochondrial function by dietary interventions, at least as far as curcumin is concerned [109]. In contrast, supplementation of acetyl-L-carnitine and lipoic acid, the latter one being the coenzyme of mitochondrial pyruvate dehydrogenase and α-ketoglutarate dehydrogenase, led to cognitive improvements in APOE4 TR mice [110]. It should be noted that both acetyl-L-carnitine and lipoic acid are also synthesized endogenously; thus responsiveness of *APOE ε4* to dietary factors may depend on the origin of the substances. Furthermore, differential modulation of mitochondrial functions seems to depend on the cell types investigated; an observation that may not be limited to the brain but may also be applicable to other organs and tissues. Table 2 provides an overview of studies with relevant findings concerning the association of the *APOE* genotype with mitochondrial function.

**Table 2:** Selected studies in which the effect of the *APOE* genotype on mitochondrial function-related traits was investigated. Only studies were included in which effects of APOE4 were compared with those of APOE3

Reference	Model system	Main findings; APOE4 vs. APOE3
Mosconi et al. [88, 89]; Langbaum et al. [90]; Small et al. [91, 93]; Reiman et al. [92, 94–96]	Human subjects with and without diagnosed AD (brain)	-Glucose hypometabolism
Xu et al. [105]	Hippocampi of human *APOE ε3/ε3* and *APOE ε4/ε4* AD patients	-Mitochondrial oxidative phosphorylation and energy metabolism ↓
Turchan-Cholewo et al. [173]	Human neuronal cultures	-GSH concentrations and mitochondrial membrane potential after treatment with HIV protein and opiate ↓ No effect in astrocytes.
Valla et al. [174]	Young *APOE ε4* carriers and non-carriers (posterior cingulate cortex tissue)	-CIV activity ↓
Chen et al. [87]	Primary neurons from NSE-APOE transgenic mice (cortex) Primary astrocytes from GFAP-APOE transgenic mice (cortex) Neuro-2A cells stably expressing APOE3 and APOE4, resp.	-Protein levels CI-V ↓ -Protein levels CI-V ↔ -Protein levels CI, CIV and CV ↓ -mRNA expression CIV subunit 1, CV subunit α ↓, Vdac1 ↑ -Activity CIV↓, citrate synthase ↔ -Mitochondrial respiratory capacity ↓ APOE4-R61T mutation or GIND24 treatment abolished detrimental effects on mitochondria.
James et al. [106]	Mitochondrial fractions (hippocampus) from APOE transgenic mice	-Differential regulation of TCA cycle, ETC and ATP synthesis
Liraz et al. [108]	Young APOE TR mice (4 months old, hippocampal neurons)	-Protein expression Tomm40, CIV subunit 1 ↓
Chin et al. [109]	Old APOE TR mice (15 months old, cortex)	-ATP levels ↓ -mRNA expression Ppargc1a, Gabpa ↓
Shi et al. [107]	Mitochondrial fractions (synaptosomes) from GFAP-APOE transgenic mice	-Pathways TCA, ETC, oxidation reduction ↓ -ATP synthesis ↓ -GSSG, MDA ↑ Effect stronger in presence of female gender. No effect in non-synaptic mitochondria.
Tambini et al. [175]	Human fibroblasts treated with astrocyte-conditioned media obtained from APOE4 and APOE3 TR mice, resp.	-MAM activity ↑

*APOE* apolipoprotein E, *ATP* adenosine triphosphate, *CI-V* complexes I-V of the mitochondrial respiratory chain, *ETC* electron transport chain, *Gabpa* GA-binding protein alpha chain, *GSH* glutathione, *GSSG* glutathione disulfide, *HIV* human immunodeficiency virus, *MAM* mitochondria-associated endoplasmic reticulum (ER) membranes, *MDA* malondialdehyde, *Ppargc1a*, peroxisome proliferator-activated receptor gamma, coactivator 1 alpha, *TCA* tricarboxylic acid, *Tomm40* translocase of the outer mitochondrial membrane

An association between the *APOE* genotype and mitochondrial function is further substantiated by linkage disequilibrium (LD) structure analysis of the *APOE* gene and its neighboring regions which revealed high LD levels between three SNPs in the *TOMM40* gene and the *APOE ε4* allele [111].

### *APOE* genotype and the inflammatory response

As stated earlier, all three pathways of the UPR may trigger the inflammatory response by activating the pro-inflammatory transcription factor NF-kB [74]. Furthermore, oxidative stress and mitochondrial dysfunction are implicated in the inflammatory immune response. Substances released by damaged mitochondria promote inflammation. Eventually, the heightened inflammatory response in turn worsens ER stress and mitochondrial dysfunction [112]. Noteworthy, one characteristic of the aging process is the induction of low-grade chronic inflammation, often referred to as inflammaging, which is believed to be involved in the pathogenesis of several age-related chronic diseases, including AD and CVD [113]. An increasing number of evidence supports an APOE isoform-dependent modulation of the inflammatory response, with APOE4 appearing to be more associated with an overactive pro-inflammatory response to diverse stimuli (reviewed in [55]). In a number of studies, APOE4 has been shown to be less effective in down-regulating the activation of microglia and peripheral macrophages and suppressing the release of pro-inflammatory cytokines and other inflammatory mediators both in vitro and in vivo [114–118]. On the contrary, two studies reported lower levels of pro-inflammatory cytokines in APOE4 vs. APOE3 expressing astrocytes [119] and Schwann cells (the principal glia cells in the CNS) [120]. The authors discuss this as a sign of APOE4-dysfunctionality in the production of cytokines to combat inflammation [119, 120]. Strikingly, in co-culture systems of microglia expressing the APOE isoforms and neurons, neurotoxicity was shown to be greatest with APOE4 expressing microglia [114]. Activated-like morphology of microglia in presence of APOE4 has already been observed in un-stimulated cells, potentially pointing towards a pro-inflammatory phenotype as an inherited property of APOE4 expressing cells [115]. Accordingly, in brains of AD patients, significantly more activated and scattered microglia were observed in carriers of the *ε4* allele [121]. The main signaling transduction pathways involved in the direction of the APOE isoform-specific inflammatory responses include the NF-kB and the mitogen-activated protein kinase MAPK (p38 MAPK and JNK) pathways [114, 119, 122]. Very recent evidence indicates that APOE4 not only enhances pro-inflammatory pathways, but may also impact on the inflammatory response via the suppression of anti-inflammatory pathways. While NF-kB activation and the subsequent prostaglandin E2 (PGE2)-pathway were significantly up-regulated in primary microglia of APOE4 vs. APOE3 TR mice, the expression of the anti-inflammatory *TREM2* (triggering receptor expressed on myeloid cells 2) was suppressed in presence of APOE4 [123]. However, it should be noted that, though the heightened inflammatory response in presence of *APOE ε4* may be deleterious to the organism with advanced age, it may confer greater protection from pathogens early in life (“antagonistic pleiotropy” theory, [124]). Indeed, a still quite high prevalence of *APOE ε4* is still observed in certain indigenous populations, including Pygmies, Khoisan, and aborigines of Malaysia and Australia, where *APOE ε4* frequency ranges from 24 to 40.7 % [125]. Interestingly, spontaneous abortions [126] and still-births [127] were found to be less frequent in *APOE ε4* allele carriers, suggesting that APOE4 somehow protects the developing embryo [126, 127]. Furthermore, Oriá et al. [128] reported positive effects of *APOE ε4* on the cognitive and physical development in children who suffered from heavy diarrhea in early childhood in a slum region in Brazil, where malnutrition is common [128–130]. Moreover, *APOE ε4* appeared beneficial in certain infectious diseases evoked by both viruses (e.g., hepatitis C, HCV) and bacteria (e.g., malaria) [131–133], though, on the contrary seemed rather detrimental in the case of human immunodeficiency virus (HIV) and herpes simplex virus (HSV) infections [134, 135] (reviewed in [136]).

## Therapeutic approaches targeting APOE4

Nowadays, several companies offer genetic testing to the public and provide their customers with information about ancestry, disease susceptibility genes, and about effects of dietary habits, drug use and other environmental factors on the individuals’ health. Websites offering information and support for people genotyped positive for certain gene variants arise quickly. The *APOE* gene is gaining more and more public attention. The role of APOE4 as a mortality factor in the elderly is undisputed. Because of the magnitude of impact of APOE4 on various age-related diseases and the relatively high frequency of the *ε4* allele in the population, prevention strategies targeting APOE4 gain in importance, particularly in view of the ongoing “population aging” [137]. Several approaches come into consideration, including pharmacoprevention strategies, dietary and lifestyle interventions, as well as gene editing.

### Pharmacoprevention

#### APOE inducers

One target of interest in pharmacoprevention research is the discovery of drugs that raise APOE levels. *APOE ε4* carriers have been shown to display lower APOE levels in blood [138–141] and brain tissue [141–144]. Interestingly, the *ε2* allele, whose carriers seem more likely to reach centenarian age, is associated with the highest blood APOE levels among the APOE isoforms [145]. Few candidate drugs that induce APOE production have been identified so far. The non-steroidal anti-inflammatory drugs indomethacin and aspirin induced APOE levels in rat microglia and astrocyte cell cultures via an unknown mechanism [146]. Probucol, a cholesterol-lowering drug, induced APOE and low density lipoprotein receptor-related protein (LRP) in the hippocampus of aged rats [147]. Agonists of the liver X receptor (LXR) and retinoid X receptor (RXR) may also be promising candidates. Both have been shown to induce APOE production on the transcript level [145, 148, 149]. Furthermore, 17ß-estradiol elevated APOE protein levels in mice [150]. Indeed, some detrimental effects of APOE4 have been shown preventable by the administration of APOE-inducers. Bexarotene for example, an RXR agonist, raised hope, when Cramer et al. [149] reported it to enhance amyloid beta (Aß)-clearance and to restore cognitive deficits in a mouse model of AD, though, since then, increasing evidence queries the effectiveness of bexarotene in preventing AD characteristic features [151, 152]. The increase in APOE levels induced by probucol was shown to improve Alzheimer’s Disease Assessment Scale-Cognitive subscale (ADAS-Cog) scores and to inversely correlate with phosphorylated tau in the cerebrospinal fluid (CSF), a marker of neuronal damage in AD subjects [145]. Next to APOE-inducing agents, APOE mimetic peptides are under investigation for the treatment of pathological changes in the central nervous system, and potentially these peptides can eventually also be administered to compensate APOE4 functional deficits [153, 154]. It should be noted that opposing evidence exists, suggesting that reducing rather than increasing APOE expression might be the more preferable approach, at least as far as the lowering of brain Aβ levels is concerned [155]. The reassessment of drugs which are already on the market for other purposes might be a promising strategy to discover new treatment opportunities for detrimental health outcomes associated with the *ε4* allele.

#### Small-molecule structure correctors

Another pharmacotherapeutic approach, though still in its infancy, is the search for so-called small-molecule structure correctors, i.e., substances that induce correct APOE4 folding to restore proper protein function [137]. As mentioned previously, it was proposed that APOE4 is structurally different from APOE3 and APOE2 in a way that allows an interaction between the amino- and carboxyl-terminal domain [45]. Whether this interaction is based on a salt bridge between two amino acid residues or is rather attributable to allosteric effects remains to be clarified [47], as stated earlier. In addition to this so-called “domain interaction”, APOE4 has been shown to assume the form of a molten globule [44]. Researchers from the Gladstone Institute in San Francisco, US, who established a screening platform for putative structure correctors, are forefront in the discovery of substances able to convert APOE4 into an APOE3-like conformation. Readouts cover both APOE4 intramolecular interaction (or the disruption thereof; fluorescence resonance energy transfer (FRET) assay) and diverse assays to measure functional effects, mainly involving cell culture systems [137]. Few compounds with high potency (nanomolar range) as putative APOE4 structure correctors, mainly phthalazinone analogs, have been identified so far and have proved effectiveness in vitro. Treatment of APOE4 with these substances restored mt-CO1 (CIV) expression and mitochondrial motility, and promoted neurite outgrowth in cultured neurons [156]. Earlier identified substances with lower potency (millimolar range) had already been shown to restore normal trafficking in the endoplasmic reticulum and Golgi apparatus [76] and to decrease Aß-production [157] in vitro. However, structure correctors identified so far have caused toxicity in mice. A pyrazoline analogue identified more recently may eventually hold some promise [156].

### Dietary and lifestyle strategies

Pharmacoprevention may be the most effective strategy to ameliorate detrimental outcomes associated with APOE4; however, the discovery of new drugs is time-consuming and cost-intensive. Furthermore, drug discovery trials often come to nothing, when safety concerns are encountered. Therefore, alternative strategies become more and more important, including dietary and lifestyle interventions [145]. Studies investigating the effects of dietary fat manipulation on blood lipid levels in relation to the *APOE* genotype yielded inconsistent results. However, *APOE* variation may influence the effects of fish oil supplementation on blood LDL levels. In a group of individuals with an atherogenic lipoprotein phenotype, *APOE ε4* carriers showed a greater raise in LDL cholesterol levels after fish oil supplementation (eicosapentaenoic acid (EPA) and docosahexaenoic acid (DHA), 3 g/d) compared with carriers of the other APOE isoforms [158], though the effect may be highly dose-dependent [159]. As aforementioned, *APOE ε4* appeared rather insensitive to other dietary interventions. Quercetin supplementation, for example, exerted beneficial effects (blood pressure and plasma tumor necrosis factor alpha (TNF-α) reduction) only in presence of *APOE ε3*, but not *APOE ε4* in humans and APOE TR mice [160, 161]. Similarly, curcumin supplementation raised ATP levels only in APOE3, but not in APOE4 mice [109]. However, supplementation of acetyl-L-carnitine and lipoic acid was shown to improve cognitive functions in APOE4 TR mice [110], as stated earlier. *APOE ε4* carriers may have a slightly increased demand for dietary vitamin E, since vitamin E uptake into extrahepatic tissues was shown to be reduced and vitamin E degradation enhanced in APOE4 compared with APOE3 mice [162–164]. On the other hand, *APOE ε4* carriers were shown to exhibit higher intestinal absorption of dietary vitamin D and calcium [165]. *APOE ε4* carriers can also benefit from increasing their physical activity. It was shown that physical activity reduced the risk of CVD and protected cognitive functions especially in carriers of the *ε4* allele [166, 167]. However, success of lifestyle interventions depends on the collaboration of the people concerned, what ultimately questions real-life-applicability [145].

### Genome editing

New therapeutic possibilities may arise from the use of ‘genome editing’, i.e., the direct manipulation of the DNA sequence. Initial methods aiming at a conversion of *APOE ε4* into *APOE ε3* were based on homologous recombination [36]. First in vitro approaches using hybrid RNA-DNA oligonucleotides, so-called chimeraplasts, were shown to cause cytotoxic side-effects [168]. Improved methods using shorter single-stranded all-DNA oligonucleotides have appeared more promising so far [36]. Substantial progress in the field of gene editing in general was made by the use of specific nucleases that can cleave the DNA at nearly every desired position including ZFNs (zinc-finger nucleases), TALENs (transcription activator-like effector nucleases) and the CRISPR/Cas9 (Clustered Regularly Interspaced Short Palindromic Repeats/Cas9 nuclease) system [169]. It remains to be established whether these tools will eventually be used to target the genomic sequence of *APOE ε4* [36].

## Conclusion and outlook

Taken together, the current review highlights the impact of the *APOE* genotype on the modulation of stress response processes. However, the findings reviewed within this work support tissue-specific pathological patterns of APOE4. In the light of the pleiotropic nature of the APOE protein, therapeutic interventions targeting APOE4 should be chosen carefully not to abolish beneficial features of APOE4. As mentioned earlier, APOE4 may be beneficial early in life when infectious diseases are of greater importance to health than chronic disorders. This may especially apply to populations under high infectious pressure and a high prevalence of malnutrition. Furthermore, we previously reported associations of *APOE ε4* with higher levels of both vitamin D [165] and adiponectin [170]. Strikingly, there is also evidence for *APOE ε4* being protective in age-related macular degeneration, a classical age-related disease [171]. Nevertheless, in industrialized countries, APOE4 is associated with premature death. Although the mechanisms by which APOE4 exerts its pathologic effects remain largely unknown, a recent study showing nuclear translocation and direct transcriptional activity of APOE4 with binding sites in promoter regions of as many as 1700 genes (among others, genes involved in neuron nourishment, cell death and aging) [172] opens up new possibilities on the mode of action of APOE4. This knowledge may potentially help targeting and treating effects of APOE4 more specifically.

## Abbreviations

AD, Alzheimer’s disease; ADAS-Cog, Alzheimer’s Disease Assessment Scale-Cognitive subscale; AMP, adenosine monophosphate; AMPK, adenosine monophosphate-activated protein kinase; APOE, apolipoprotein E; Aß, amyloid beta; ATF4, activating transcription factor 4, ATF6, activating transcription factor 6; ATG7, autophagy related 7; ATP, adenosine triphosphate; BCL2, B cell lymphoma 2 family members; BIP, binding immunoglobulin protein; CGAS, candidate gene association study; CHD, coronary heart disease; CHOP, c/EBP-homologous protein 10; CI-V, complexes I-V of the mitochondrial respiratory chain; CM, chylomicron; CMR, chylomicron remnant; CRISPR/Cas9, Clustered Regularly Interspaced Short Palindromic Repeats/Cas9 nuclease; CSF, cerebrospinal fluid; CVD, cardiovascular disease; DDIT3, DNA damage-inducible transcript 3; DNM1L or DRP1, dynamin 1-like protein 1; EGFP, enhanced green fluorescent protein; EIF2A, eukaryotic translation initiation factor 2A; ER, endoplasmic reticulum; ETC, electron transport chain; FFA, free fatty acids; FOXN3, forkhead box N3; FOXO3, forkhead box O3; FRET, fluorescence resonance energy transfer; Gabpa, GA-binding protein alpha chain; GADD34, protein phosphatase 1, regulatory subunit 15A; GCN, eukaryotic translation initiation factor 2 alpha kinase 4; GRP78, 78 kDa glucose-regulated protein; GRP94, heat shock protein 90, beta; GSH, glutathione; GSSG, glutathione disulfide; GWAS, genome-wide association study; HCV, hepatitis C virus; HDL, high density lipoprotein; HERP, homocysteine-inducible, endoplasmic reticulum stress-inducible, ubiquitin-like domain member 1; HIV, human immunodeficiency virus; HL, hepatic lipase; HSPG, heparan sulfate proteoglycans; HSV, herpes simplex virus; IP3R, inositol 1,4,5-triphosphate receptor; IRE1α, serine/threonine-protein kinase/endoribonuclease; JNK, mitogen-activated protein kinase 8; KO, knockout; LD, linkage disequilibrium; LDL, low density lipoprotein; LDLR, low density lipoprotein receptor; LPL, lipoprotein lipase; LRP, low density lipoprotein receptor-related protein; LXR, liver X receptor; MAM, mitochondria-associated endoplasmic reticulum membranes; MAPK, mitogen-activated protein kinase; MDA, malondialdehyde; MFN1/2, mitofusin 1/2; mt-CO1 or CIV, mitochondrial cytochrome oxidase I; NRF, nuclear respiratory factors; OASIS, old astrocyte specifically induced substance; OPA1, optic atrophy 1 protein; OXPHOS, oxidative phosphorylation; PDI, protein disulfide isomerase associated 3; PERK, eukaryotic translation initiation factor 2-alpha kinase 3; PET, positron-emission tomography; PGE2, prostaglandin E2; PKR, eukaryotic translation initiation factor 2-alpha kinase 2; PON1, paraoxonase 1; PPAR, peroxisome proliferator-activated receptors; Ppargc1a or PGC-1α, peroxisome proliferator-activated receptor gamma, coactivator 1 alpha; RCT, reverse cholesterol transport; ROS, reactive oxygen species; RXR, retinoid X receptor; SIRT1, sirtuin 1; TALEN, transcription activator-like effector nucleases; TCA, tricarboxylic acid; TNF-α, tumor necrosis factor alpha; TOMM40, translocase of the outer mitochondrial membrane; TR, targeted replacement; TRB3, tribbles homolog 3; TREM2, triggering receptor expressed on myeloid cells 2; UBA5, ubiquitin like modifier activating enzyme 5; UPR, unfolded protein response; VDAC1, voltage-dependent anion channel 1; VLDL, very low density lipoprotein; XBP1(S), X-box binding protein 1, spliced; XBP1, X-box binding protein 1; ZFN, zinc-finger nucleases

## References

[1] Kregel KC, Zhang HJ (2007). An integrated view of oxidative stress in aging: basic mechanisms, functional effects, and pathological considerations. Am J Physiol Regul Integr Comp Physiol..

[2] Vasto S, Candore G, Balistreri CR, Caruso M, Colonna-Romano G, Grimaldi MP, Listi F, Nuzzo D, Lio D, Caruso C (2007). Inflammatory networks in ageing, age-related diseases and longevity. Mech Ageing Dev..

[3] Harman D (2001). Aging: overview. Ann N Y Acad Sci..

[4] McGue M, Vaupel JW, Holm N, Harvald B (1993). Longevity is moderately heritable in a sample of Danish twins born 1870–1880. J Gerontol..

[5] Herskind AM, McGue M, Holm NV, Sørensen TIA, Harvald B, Vaupel JW (1996). The heritability of human longevity: a population-based study of 2872 Danish twin pairs born 1870–1900. Hum Genet..

[6] Hjelmborg J, Iachine I, Skytthe A, Vaupel JW, McGue M, Koskenvuo M, Kaprio J, Pedersen NL, Christensen K (2006). Genetic influence on human lifespan and longevity. Hum Genet..

[7] Christensen K, Johnson TE, Vaupel JW (2006). The quest for genetic determinants of human longevity: challenges and insights. Nat Rev Genet..

[8] Broer L, Buchman AS, Deelen J, Evans DS, Faul JD, Lunetta KL, Sebastiani P, Smith JA, Smith AV, Tanaka T, Yu L, Arnold AM, Aspelund T, Benjamin EJ, De Jager PL, Eirkisdottir G, Evans DA, Garcia ME, Hofman A, Kaplan RC, Kardia SLR, Kiel DP, Oostra BA, Orwoll ES, Parimi N, Psaty BM, Rivadeneira F, Rotter JI, Seshadri S, Singleton A, Tiemeier H, Uitterlinden AG, Zhao W, Bandinelli S, Bennett DA, Ferrucci L, Gudnason V, Harris TB, Karasik D, Launer LJ, Perls TT, Slagboom PE, Tranah GJ, Weir DR, Newman AB, van Duijn CM, Murabito JM (2015). GWAS of longevity in CHARGE consortium confirms APOE and FOXO3 candidacy. J Gerontol A Biol Sci Med Sci.

[9] Schächter F, Faure-Delanef L, Guénot F, Rouger H, Froguel P, Lesueur-Ginot L, Cohen D (1994). Genetic associations with human longevity at the APOE and ACE loci. Nat Genet..

[10] Jian-Gang Z, Yong-Xing M, Chuan-Fu W, Pei-Fang L, Song-Bai Z, Nui-Fan G, Guo-Yin F, Lin H (1998). Apolipoprotein E and longevity among Han Chinese population. Mech Ageing Dev..

[11] Blanché H, Cabanne L, Sahbatou M, Thomas G (2001). A study of French centenarians: are ACE and APOE associated with longevity?. C R Acad Sci III..

[12] Flachsbart F, Caliebe A, Kleindorp R, Blanché H, von Eller-Eberstein H, Nikolaus S, Schreiber S, Nebel A (2009). Association of FOXO3A variation with human longevity confirmed in German centenarians. Proc Natl Acad Sci U S A.

[13] Nebel A, Kleindorp R, Caliebe A, Nothnagel M, Blanché H, Junge O, Wittig M, Ellinghaus D, Flachsbart F, Wichmann HE, Meitinger T, Nikolaus S, Franke A, Krawczak M, Lathrop M, Schreiber S (2011). A genome-wide association study confirms APOE as the major gene influencing survival in long-lived individuals. Mech Ageing Dev..

[14] Deelen J, Beekman M, Uh HW, Helmer Q, Kuningas M, Christiansen L, Kremer D, van der Breggen R, Suchiman HED, Lakenberg N, van den Akker EB, Passtoors WM, Tiemeier H, van Heemst D, de Craen AJ, Rivadeneira F, de Geus EJ, Perola M, van der Ouderaa FJ, Gunn DA, Boomsma DI, Uitterlinden AG, Christensen K, van Duijn CM, Heijmans BT, Houwing-Duistermaat JJ, Westendorp RG, Slagboom PE (2011). Genome-wide association study identifies a single major locus contributing to survival into old age; the APOE locus revisited. Aging Cell.

[15] Fuzikawa AK, Peixoto SV, Taufer M, Moriguchi EH, Lima-Costa MF (2007). Apolipoprotein E polymorphism distribution in an elderly Brazilian population: the Bambuí Health and Aging Study. Brazilian J Med Biol Res..

[16] Mahley RW (1988). Apolipoprotein E : cholesterol transport protein with expanding role in cell biology. Science.

[17] Williams DL, Dawson PA, Newman TC, Rudel LL (1985). Apolipoprotein E synthesis in peripheral tissues of nonhuman primates. J Biol Chem..

[18] Getz GS, Reardon CA (2009). Apoprotein E as a lipid transport and signaling protein in the blood, liver, and artery wall. J Lipid Res..

[19] Ang LS, Cruz RP, Hendel A, Granville DJ (2008). Apolipoprotein E, an important player in longevity and age-related diseases. Exp Gerontol..

[20] Eichner JE, Dunn ST, Perveen G, Thompson DM, Stewart KE, Stroehla BC (2002). Apolipoprotein E polymorphism and cardiovascular disease: a HuGE review. Am J Epidemiol..

[21] Weisgraber KH (1994). Apolipoprotein E: structure-function relationships. Adv Protein Chem.

[22] Matsuura F, Wang N, Chen W, Jiang XC, Tall AR (2006). HDL from CETP-deficient subjects shows enhanced ability to promote cholesterol efflux from macrophages in an apoE- and ABCG1-dependent pathway. J Clin Invest..

[23] Zanotti I, Pedrelli M, Potì F, Stomeo G, Gomaraschi M, Calabresi L, Bernini F (2011). Macrophage, but not systemic, apolipoprotein E is necessary for macrophage reverse cholesterol transport in vivo. Arterioscler Thromb Vasc Biol..

[24] Piedrahita JA, Zhang SH, Hagaman JR, Oliver PM, Maeda N (1992). Generation of mice carrying a mutant apolipoprotein E gene inactivated by gene targeting in embryonic stem cells. Proc Natl Acad Sci U S A.

[25] Moghadasian MH, McManus BM, Nguyen LB, Shefer S, Nadji M, Godin DV, Green TJ, Hill J, Yang Y, Scudamore CH, Frohlich JJ (2001). Pathophysiology of apolipoprotein E deficiency in mice: relevance to apo E-related disorders in humans. FASEB J..

[26] Zhang SH, Reddick RL, Piedrahita JA, Maeda N (1992). Spontaneous hypercholesterolemia and arterial lesions in mice lacking apolipoprotein E. Science.

[27] Robertson TA, Dutton NS, Martins RN, Taddei K, Papadimitriou JM (2000). Comparison of astrocytic and myocytic metabolic dysregulation in apolipoprotein E deficient and human apolipoprotein E transgenic mice. Neuroscience.

[28] Roselaar SE, Daugherty A (1998). Apolipoprotein E-deficient mice have impaired innate immune responses to Listeria monocytogenes in vivo. J Lipid Res..

[29] de Bont N, Netea MG, Demacker PN, Verschueren I, Kullberg BJ, van Dijk KW, van der Meer JW, Stalenhoef AF (1999). Apolipoprotein E knock-out mice are highly susceptible to endotoxemia and Klebsiella pneumoniae infection. J Lipid Res..

[30] Van Oosten M, Rensen PCN, Van Amersfoort ES, Van Eck M, Van Dam A-M, Brevé JJP, Vogel T, Panet A, Van Berkel TJC, Kuiper J (2001). Apolipoprotein E protects against bacterial lipopolysaccharide-induced lethality. A new therapeutic approach to treat gram-negative sepsis. J Biol Chem.

[31] Hayek T, Oiknine J, Brook JG, Aviram M (1994). Increased plasma and lipoprotein lipid peroxidation in apo E-deficient mice. Biochem Biophys Res Commun..

[32] Miyata M, Smith JD (1996). Apolipoprotein E allele-specific antioxidant activity and effects on cytotoxicity by oxidative insults and beta-amyloid peptides. Nat Genet..

[33] Ikeno Y (2015). New insights and current concepts of the oxidative stress theory of aging. Arch Biochem Biophys..

[34] Tarnus E, Wassef H, Carmel JF, Rondeau P, Roche M, Davignon J, Bernier L, Bourdon E (2009). Apolipoprotein E limits oxidative stress-induced cell dysfunctions in human adipocytes. FEBS Lett..

[35] Lusis AJ, Heinzmann C, Sparkes RS, Scott J, Knott TJ, Geller R, Sparkes MC, Mohandas T (1986). Regional mapping of human chromosome 19: organization of genes for plasma lipid transport (APOC1, -C2, and -E and LDLR) and the genes C3, PEPD, and GPI. Proc Natl Acad Sci U S A.

[36] Papaioannou I, Simons JP, Owen JS (2012). Targeted in situ gene correction of dysfunctional APOE alleles to produce atheroprotective plasma ApoE3 protein. Cardiol Res Pract..

[37] Lee Y, Kockx M, Raftery MJ, Jessup W, Griffith R, Kritharides L (2010). Glycosylation and sialylation of macrophage-derived human apolipoprotein E analyzed by SDS-PAGE and mass spectrometry: evidence for a novel site of glycosylation on Ser290. Mol Cell Proteomics.

[38] Weisgraber KH, Rall SC, Mahley RW (1981). Human E apoprotein heterogeneity. Cysteine-arginine interchanges in the amino acid sequence of the apo-E isoforms. J Biol Chem.

[39] Hatters DM, Peters-Libeu CA, Weisgraber KH (2006). Apolipoprotein E structure: insights into function. Trends Biochem Sci..

[40] Singh PP, Singh M, Mastana SS (2006). APOE distribution in world populations with new data from India and the UK. Ann Hum Biol..

[41] Frieden C, Garai K (2012). Structural differences between apoE3 and apoE4 may be useful in developing therapeutic agents for Alzheimer’s disease. Proc Natl Acad Sci U S A.

[42] Dong LM, Wilson C, Wardell MR, Simmons T, Mahley RW, Weisgraber KH, Agard DA (1994). Human apolipoprotein E. Role of arginine 61 in mediating the lipoprotein preferences of the E3 and E4 isoforms. J Biol Chem.

[43] Acharya P, Segall ML, Zaiou M, Morrow J, Weisgraber KH, Phillips MC, Lund-Katz S, Snow J (2002). Comparison of the stabilities and unfolding pathways of human apolipoprotein E isoforms by differential scanning calorimetry and circular dichroism. Biochim Biophys Acta.

[44] Morrow JA, Hatters DM, Lu B, Höchtl P, Oberg KA, Rupp B, Weisgraber KH (2002). Apolipoprotein E4 forms a molten globule: A potential basis for its association with disease. J Biol Chem..

[45] Dong LM, Weisgraber KH (1996). Human apolipoprotein E4 domain interaction. Arginine 61 and glutamic acid 255 interact to direct the preference for very low density lipoproteins. J Biol Chem.

[46] Chen J, Li Q, Wang J (2011). Topology of human apolipoprotein E3 uniquely regulates its diverse biological functions. Proc Natl Acad Sci U S A.

[47] Mizuguchi C, Hata M, Dhanasekaran P, Nickel M, Okuhira K, Phillips MC, Lund-Katz S, Saito H (1841). Fluorescence study of domain structure and lipid interaction of human apolipoproteins E3 and E4. Biochim Biophys Acta.

[48] Williams B, Convertino M, Das J, Dokholyan NV (2015). ApoE4-specific misfolded intermediate identified by molecular dynamics simulations. PLoS Comput Biol..

[49] Nguyen D, Dhanasekaran P, Nickel M, Mizuguchi C, Watanabe M, Saito H, Phillips MC, Lund-Katz S (2014). Influence of domain stability on the properties of human apolipoprotein E3 and E4 and mouse apolipoprotein E. Biochemistry.

[50] Weisgraber KH, Innerarity TL, Mahley RW (1982). Abnormal lipoprotein receptor-binding activity of the human E apoprotein due to cysteine-arginine interchange at a single site. J Biol Chem..

[51] Corder EH, Saunders AM, Strittmatter WJ, Schmechel DE, Gaskell PC, Small GW, Roses AD, Haines JL, Pericak-Vance MA (1993). Gene dose of apolipoprotein E type 4 allele and the risk of Alzheimer’s disease in late onset families. Science.

[52] McKay GJ, Silvestri G, Chakravarthy U, Dasari S, Fritsche LG, Weber BH, Keilhauer CN, Klein ML, Francis PJ, Klaver CC, Vingerling JR, Ho L, De Jong PTDV, Dean M, Sawitzke J, Baird PN, Guymer RH, Stambolian D, Orlin A, Seddon JM, Peter I, Wright AF, Hayward C, Lotery AJ, Ennis S, Gorin MB, Weeks DE, Kuo CL, Hingorani AD, Sofat R, Cipriani V, Swaroop A, Othman M, Kanda A, Chen W, Abecasis GR, Yates JR, Webster AR, Moore AT, Seland JH, Rahu M, Soubrane G, Tomazzoli L, Topouzis F, Vioque J, Young IS, Fletcher AE, Patterson CC (2011). Variations in apolipoprotein e frequency with age in a pooled analysis of a large group of older people. Am J Epidemiol.

[53] Gerdes LU, Jeune B, Ranberg KA, Nybo H, Vaupel JW (2000). Estimation of apolipoprotein E genotype-specific relative mortality risks from the distribution of genotypes in centenarians and middle-aged men: apolipoprotein E gene is a “frailty gene”, not a “longevity gene”. Genet Epidemiol..

[54] Stephens JW, Bain SC, Humphries SE (2008). Gene-environment interaction and oxidative stress in cardiovascular disease. Atherosclerosis.

[55] Jofre-Monseny L, Minihane AM, Rimbach G (2008). Impact of apoE genotype on oxidative stress, inflammation and disease risk. Mol Nutr Food Res..

[56] Pocernich CB, Sultana R, Hone E, Turchan J, Martins RN, Calabrese V, Nath A, Butterfield DA (2004). Effects of apolipoprotein E on the human immunodeficiency virus protein Tat in neuronal cultures and synaptosomes. J Neurosci Res..

[57] Ramassamy C, Averill D, Beffert U, Bastianetto S, Theroux L, Lussier-Cacan S, Cohn JS, Christen Y, Davignon J, Quirion R, Poirier J (1999). Oxidative damage and protection by antioxidants in the frontal cortex of Alzheimer’s disease is related to the apolipoprotein E genotype. Free Radic Biol Med..

[58] Jofre-Monseny L, de Pascual-Teresa S, Plonka E, Huebbe P, Boesch-Saadatmandi C, Minihane AM, Rimbach G (2007). Differential effects of apolipoprotein E3 and E4 on markers of oxidative status in macrophages. Br J Nutr..

[59] Smith JD, Miyata M, Poulin SE, Neveux LM, Craig WY (1998). The relationship between apolipoprotein e and serum oxidation-related variables is apolipoprotein e phenotype dependent. Int J Clin Lab Res..

[60] Jofre-Monseny L, Huebbe P, Stange I, Boesch-Saadatmandi C, Frank J, Jackson K, Minihane A-M, Rimbach G (2008). Influence of apolipoprotein E genotype and dietary alpha-tocopherol on redox status and C-reactive protein levels in apolipoprotein E3 and E4 targeted replacement mice. Br J Nutr..

[61] Humphries SE, Talmud PJ, Hawe E, Bolla M, Day IN, Miller GJ (2001). Apolipoprotein E4 and coronary heart disease in middle-aged men who smoke: a prospective study. Lancet.

[62] Talmud PJ, Stephens JW, Hawe E, Demissie S, Cupples LA, Hurel SJ, Humphries SE, Ordovas JM (2005). The significant increase in cardiovascular disease risk in APOE ε4 carriers is evident only in men who smoke: potential relationship between reduced antioxidant status and ApoE4. Ann Hum Genet..

[63] Dietrich M, Hu Y, Block G, Olano E, Packer L, Morrow JD, Hudes M, Abdukeyum G, Rimbach G, Minihane AM (2005). Associations between apolipoprotein E genotype and circulating F2-isoprostane levels in humans. Lipids.

[64] Holmes MV, Frikke-Schmidt R, Melis D, Luben R, Asselbergs FW, Boer JMA, Cooper J, Palmen J, Horvat P, Engmann J, Li KW, Onland-Moret NC, Hofker MH, Kumari M, Keating BJ, Hubacek JA, Adamkova V, Kubinova R, Bobak M, Khaw KT, Nordestgaard BG, Wareham N, Humphries SE, Langenberg C, Tybjaerg-Hansen A, Talmud PJ (2014). A systematic review and meta-analysis of 130,000 individuals shows smoking does not modify the association of APOE genotype on risk of coronary heart disease. Atherosclerosis.

[65] Ramassamy C, Averill D, Beffert U, Theroux L, Lussier-Cacan S, Cohn JS, Christen Y, Schoofs A, Davignon J, Poirier J (2000). Oxidative insults are associated with apolipoprotein E genotype in Alzheimer’s disease brain. Neurobiol Dis..

[66] Graeser AC, Boesch-Saadatmandi C, Lippmann J, Wagner AE, Huebbe P, Storm N, Höppner W, Wiswedel I, Gardemann A, Minihane AM, Döring F, Rimbach G (2011). Nrf2-dependent gene expression is affected by the proatherogenic apoE4 genotype-studies in targeted gene replacement mice. J Mol Med..

[67] Graeser AC, Huebbe P, Storm N, Höppner W, Döring F, Wagner AE, Rimbach G (2012). Apolipoprotein E genotype affects tissue metallothionein levels: studies in targeted gene replacement mice. Genes Nutr..

[68] Gaidukov L, Viji RI, Yacobson S, Rosenblat M, Aviram M, Tawfik DS (2010). ApoE induces serum paraoxonase PON1 activity and stability similar to ApoA-I. Biochemistry.

[69] Boesch-Saadatmandi C, Niering J, Minihane AM, Wiswedel I, Gardeman A, Wolffram S, Rimbach G (2010). Impact of apolipoprotein E genotype and dietary quercetin on paraoxonase 1 status in apoE3 and apoE4 transgenic mice. Atherosclerosis.

[70] Ross JM, Olson L, Coppotelli G (2015). Mitochondrial and ubiquitin proteasome system dysfunction in ageing and disease: two sides of the same coin?. Int J Mol Sci..

[71] Bravo R, Gutierrez T, Paredes F, Gatica D, Rodriguez AE, Pedrozo Z, Chiong M, Parra V, Quest AFG, Rothermel BA, Lavandero S (2012). Endoplasmic reticulum: ER stress regulates mitochondrial bioenergetics. Int J Biochem Cell Biol..

[72] Brewer JW (2014). Regulatory crosstalk within the mammalian unfolded protein response. Cell Mol Life Sci..

[73] Cao SS, Kaufman RJ (2014). Endoplasmic reticulum stress and oxidative stress in cell fate decision and human disease. Antioxid Redox Signal..

[74] Chaudhari N, Talwar P, Parimisetty A, Lefebvre d’Hellencourt C, Ravanan P (2014). A molecular web: endoplasmic reticulum stress, inflammation and oxidative stress. Front Cell Neurosci..

[75] Zhong N, Ramaswamy G, Weisgraber KH (2009). Apolipoprotein E4 domain interaction induces endoplasmic reticulum stress and impairs astrocyte function. J Biol Chem..

[76] Brodbeck J, McGuire J, Liu Z, Meyer-Franke A, Balestra ME, Jeong DE, Pleiss M, McComas C, Hess F, Witter D, Peterson S, Childers M, Goulet M, Liverton N, Hargreaves R, Freedman S, Weisgraber KH, Mahley RW, Huang Y (2011). Structure-dependent impairment of intracellular apolipoprotein E4 trafficking and its detrimental effects are rescued by small-molecule structure correctors. J Biol Chem..

[77] Segev Y, Michaelson DM, Rosenblum K (2013). ApoE ϵ4 is associated with eIF2α phosphorylation and impaired learning in young mice. Neurobiol Aging.

[78] Eberlé D, Kim RY, Luk FS, De Mochel NSR, Gaudreault N, Olivas VR, Kumar N, Posada JM, Birkeland AC, Rapp JH, Raffai RL (2012). Apolipoprotein E4 domain interaction accelerates diet-induced atherosclerosis in hypomorphic Arg-61 Apoe mice. Arterioscler Thromb Vasc Biol..

[79] Cash JG, Kuhel DG, Basford JE, Jaeschke A, Chatterjee TK, Weintraub NL, Hui DY (2012). Apolipoprotein E4 impairs macrophage efferocytosis and potentiates apoptosis by accelerating endoplasmic reticulum stress. J Biol Chem..

[80] Sabaretnam T, Harris MJ, Kockx M, Witting PK, Le Couteur DG, Kritharides L (2009). Effects of hydrogen peroxide and apolipoprotein E isoforms on apolipoprotein E trafficking in HepG2 cells. Clin Exp Pharmacol Physiol..

[81] Simpson JE, Ince PG, Shaw PJ, Heath PR, Raman R, Garwood CJ, Gelsthorpe C, Baxter L, Forster G, Matthews FE, Brayne C, Wharton SB (2011). Microarray analysis of the astrocyte transcriptome in the aging brain: relationship to Alzheimer’s pathology and APOE genotype. Neurobiol Aging.

[82] Nunnari J, Suomalainen A (2012). Mitochondria: in sickness and in health. Cell.

[83] Lopez-Mejia IC, Fajas L (2015). Cell cycle regulation of mitochondrial function. Curr Opin Cell Biol..

[84] Nicholls DG (2002). Mitochondrial function and dysfunction in the cell: its relevance to aging and aging-related disease. Int J Biochem Cell Biol..

[85] Kaufman RJ, Malhotra JD (1843). Calcium trafficking integrates endoplasmic reticulum function with mitochondrial bioenergetics. Biochim Biophys Acta.

[86] Hauptmann S, Scherping I, Dröse S, Brandt U, Schulz KL, Jendrach M, Leuner K, Eckert A, Müller WE (2009). Mitochondrial dysfunction: an early event in Alzheimer pathology accumulates with age in AD transgenic mice. Neurobiol Aging.

[87] Chen HK, Ji ZS, Dodson SE, Miranda RD, Rosenblum CI, Reynolds IJ, Freedman SB, Weisgraber KH, Huang Y, Mahley RW (2011). Apolipoprotein E4 domain interaction mediates detrimental effects on mitochondria and is a potential therapeutic target for Alzheimer disease. J Biol Chem..

[88] Mosconi L, Nacmias B, Sorbi S, De Cristofaro MTR, Fayazz M, Tedde A, Bracco L, Herholz K, Pupi A (2004). Brain metabolic decreases related to the dose of the ApoE e4 allele in Alzheimer’s disease. J Neurol Neurosurg Psychiatry.

[89] Mosconi L, Herholz K, Prohovnik I, Nacmias B, De Cristofaro MTR, Fayyaz M, Bracco L, Sorbi S, Pupi A (2005). Metabolic interaction between ApoE genotype and onset age in Alzheimer’s disease: implications for brain reserve. J Neurol Neurosurg Psychiatry.

[90] Langbaum JBS, Chen K, Caselli RJ, Lee W, Reschke C, Bandy D, Alexander GE, Burns CM, Kaszniak AW, Reeder SA, Corneveaux JJ, Allen AM, Pruzin J, Huentelman MJ, Fleisher AS, Reiman EM (2010). Hypometabolism in Alzheimer-affected brain regions in cognitively healthy Latino individuals carrying the APOE4 epsilon4 allele. Arch Neurol.

[91] Small GW, Mazziotta JC, Collins MT, Baxter LR, Phelps ME, Mandelkern MA, Kaplan A, La Rue A, Adamson CF, Chang L (1995). Apolipoprotein E type 4 allele and cerebral glucose metabolism in relatives at risk for familial Alzheimer disease. Jama.

[92] Reiman EM, Caselli RJ, Yun LS, Chen K, Bandy D, Minoshima S, Thibodeau SN, Osborne D (1996). Preclinical evidence of Alzheimer’s disease in persons homozygous for the epsilon 4 allele for apolipoprotein E. N Engl J Med..

[93] Small GW, Ercoli LM, Silverman DH, Huang SC, Komo S, Bookheimer SY, Lavretsky H, Miller K, Siddarth P, Rasgon NL, Mazziotta JC, Saxena S, Wu HM, Mega MS, Cummings JL, Saunders AM, Pericak-Vance MA, Roses AD, Barrio JR, Phelps ME (2000). Cerebral metabolic and cognitive decline in persons at genetic risk for Alzheimer’s disease. Proc Natl Acad Sci U S A.

[94] Reiman EM, Chen K, Alexander GE, Caselli RJ, Bandy D, Osborne D, Saunders AM, Hardy J (2005). Correlations between apolipoprotein E epsilon4 gene dose and brain-imaging measurements of regional hypometabolism. Proc Natl Acad Sci U S A.

[95] Reiman EM, Chen K, Caselli RJ, Alexander GE, Bandy D, Adamson JL, Lee W, Cannon A, Stephan EA, Stephan DA, Papassotiropoulos A (2008). Cholesterol-related genetic risk scores are associated with hypometabolism in Alzheimer’s-affected brain regions. Neuroimage.

[96] Reiman EM, Chen K, Alexander GE, Caselli RJ, Bandy D, Osborne D, Saunders AM, Hardy J (2004). Functional brain abnormalities in young adults at genetic risk for late-onset Alzheimer’s dementia. Proc Natl Acad Sci U S A.

[97] Hoyer S (1995). Age-related changes in cerebral oxidative metabolism. Implications for drug therapy. Drugs Aging.

[98] Huang Y, Liu XQ, Wyss-Coray T, Brecht WJ, Sanan DA, Mahley RW (2001). Apolipoprotein E fragments present in Alzheimer’s disease brains induce neurofibrillary tangle-like intracellular inclusions in neurons. Proc Natl Acad Sci U S A.

[99] Harris FM, Brecht WJ, Xu Q, Tesseur I, Kekonius L, Wyss-Coray T, Fish JD, Masliah E, Hopkins PC, Scearce-Levie K, Weisgraber KH, Mucke L, Mahley RW, Huang Y (2003). Carboxyl-terminal-truncated apolipoprotein E4 causes Alzheimer’s disease-like neurodegeneration and behavioral deficits in transgenic mice. Proc Natl Acad Sci U S A.

[100] Tamboli IY, Heo D, Rebeck GW (2014). Extracellular proteolysis of apolipoprotein E (apoE) by secreted serine neuronal protease. PLoS One.

[101] Xu H, Gupta VB, Martins IJ, Martins RN, Fowler CJ, Bush AI, Finkelstein DI, Adlard PA (2015). Zinc affects the proteolytic stability of apolipoprotein E in an isoform-dependent way. Neurobiol Dis..

[102] Gonzalez C, Martin T, Cacho J, Brenas MT, Arroyo T, Garcia-Berrocal B, Navajo JA, Gonzalez-Buitrago JM (1999). Serum zinc, copper, insulin and lipids in Alzheimer’s disease epsilon 4 apolipoprotein E allele carriers. Eur J Clin Invest.

[103] Nakamura T, Watanabe A, Fujino T, Hosono T, Michikawa M (2009). Apolipoprotein E4 (1-272) fragment is associated with mitochondrial proteins and affects mitochondrial function in neuronal cells. Mol Neurodegener..

[104] Chang S, ran Ma T, Miranda RD, Balestra ME, Mahley RW, Huang Y (2005). Lipid- and receptor-binding regions of apolipoprotein E4 fragments act in concert to cause mitochondrial dysfunction and neurotoxicity. Proc Natl Acad Sci U S A.

[105] Xu PT, Li YJ, Qin XJ, Scherzer CR, Xu H, Schmechel DE, Hulette CM, Ervin J, Gullans SR, Haines J, Pericak-Vance MA, Gilbert JR (2006). Differences in apolipoprotein E3/3 and E4/4 allele-specific gene expression in hippocampus in Alzheimer disease. Neurobiol Dis..

[106] James R, Searcy JL, Le Bihan T, Martin SF, Gliddon CM, Povey J, Deighton RF, Kerr LE, McCulloch J, Horsburgh K (2012). Proteomic analysis of mitochondria in APOE transgenic mice and in response to an ischemic challenge. J Cereb Blood Flow Metab..

[107] Shi L, Du X, Zhou H, Tao C, Liu Y, Meng F, Wu G, Xiong Y, Xia C, Wang Y, Bi G, Zhou JN (2014). Cumulative effects of the ApoE genotype and gender on the synaptic proteome and oxidative stress in the mouse brain. Int J Neuropsychopharmacol..

[108] Liraz O, Boehm-Cagan A, Michaelson DM (2013). ApoE4 induces Aβ42, tau, and neuronal pathology in the hippocampus of young targeted replacement apoE4 mice. Mol Neurodegener..

[109] Chin D, Hagl S, Hoehn A, Huebbe P, Pallauf K, Grune T, Frank J, Eckert GP, Rimbach G (2014). Adenosine triphosphate concentrations are higher in the brain of APOE3- compared to APOE4-targeted replacement mice and can be modulated by curcumin. Genes Nutr..

[110] Su B, Wang X, Bonda D, Perry G, Smith M, Zhu X (2010). Abnormal mitochondrial dynamics-a novel therapeutic target for Alzheimer’s disease?. Mol Neurobiol..

[111] Yu CE, Seltman H, Peskind ER, Galloway N, Zhou PX, Rosenthal E, Wijsman EM, Tsuang DW, Devlin B, Schellenberg GD (2007). Comprehensive analysis of APOE and selected proximate markers for late-onset Alzheimer’s disease: patterns of linkage disequilibrium and disease/marker association. Genomics.

[112] Zhang K, Kaufman RJ (2008). From endoplasmic-reticulum stress to the inflammatory response. Nature.

[113] Martorana A, Bulati M, Buffa S, Pellicanò M, Caruso C, Candore G, Colonna-Romano G (2012). Immunosenescence, inflammation and Alzheimer’s disease. Longev Healthspan.

[114] Maezawa I, Nivison M, Montine KS, Maeda N, Montine TJ (2006). Neurotoxicity from innate immune response is greatest with targeted replacement of E4 allele of apolipoprotein E gene and is mediated by microglial p38MAPK. FASEB J..

[115] Vitek MP, Brown CM, Colton CA (2009). APOE genotype-specific differences in the innate immune response. Neurobiol Aging.

[116] Lynch JR, Morgan D, Mance J, Matthew WD, Laskowitz DT (2001). Apolipoprotein E modulates glial activation and the endogenous central nervous system inflammatory response. J Neuroimmunol..

[117] Ophir G, Amariglio N, Jacob-Hirsch J, Elkon R, Rechavi G, Michaelson DM (2005). Apolipoprotein E4 enhances brain inflammation by modulation of the NF-kB signaling cascade. Neurobiol Dis..

[118] Jofre-Monseny L, Loboda A, Wagner AE, Huebbe P, Boesch-Saadatmandi C, Jozkowicz A, Minihane AM, Dulak J, Rimbach G (2007). Effects of apoE genotype on macrophage inflammation and heme oxygenase-1 expression. Biochem Biophys Res Commun..

[119] Maezawa I, Maeda N, Montine TJ, Montine KS (2006). Apolipoprotein E-specific innate immune response in astrocytes from targeted replacement mice. J Neuroinflammation.

[120] Zhang KJ, Zhang HL, Zhang XM, Zheng XY, Quezada HC, Zhang D, Zhu J (2011). Apolipoprotein E isoform-specific effects on cytokine and nitric oxide production from mouse Schwann cells after inflammatory stimulation. Neurosci Lett..

[121] Egensperger R, Kösel S, von Eitzen U, Graeber MB (1998). Microglial activation in Alzheimer disease: association with APOE genotype. Brain Pathol..

[122] Zhang H, Wu LM, Wu J (2011). Cross-talk between apolipoprotein e and cytokines. Mediators Inflamm..

[123] Li X, Montine KS, Keene CD, Montine TJ (2015). Different mechanisms of apolipoprotein E isoform-dependent modulation of prostaglandin E2 production and triggering receptor expressed on myeloid cells 2 (TREM2) expression after innate immune activation of microglia. FASEB J..

[124] Finch CE (2010). Evolution in health and medicine Sackler colloquium: Evolution of the human lifespan and diseases of aging: roles of infection, inflammation, and nutrition. Proc Natl Acad Sci U S A.

[125] Trotter JH, Liebl AL, Weeber EJ, Martin LB (2011). Linking ecological immunology and evolutionary medicine: the case for apolipoprotein E. Funct Ecol..

[126] Zetterberg H, Palmér M, Ricksten A, Poirier J, Palmqvist L, Rymo L, Zafiropoulos A, Arvanitis DA, Spandidos DA, Blennow K (2002). Influence of the apolipoprotein E epsilon4 allele on human embryonic development. Neurosci Lett..

[127] Becher JC, Keeling JW, McIntosh N, Wyatt B, Bell J (2006). The distribution of apolipoprotein E alleles in Scottish perinatal deaths. J Med Genet..

[128] Oría RB, Patrick PD, Zhang H, Lorntz B, De Castro Costa CM, Brito GAC, Barrett LJ, Lima AAM, Guerrant RL (2005). APOE4 protects the cognitive development in children with heavy diarrhea burdens in Northeast Brazil. Pediatr Res..

[129] Oriá RB, Patrick PD, Blackman JA, Lima AAM, Guerrant RL (2007). Role of apolipoprotein E4 in protecting children against early childhood diarrhea outcomes and implications for later development. Med Hypotheses.

[130] Oriá RB, Patrick PD, Oriá MOB, Lorntz B, Thompson MR, Azevedo OGR, Lobo RNB, Pinkerton RF, Guerrant RL, Lima AAM (2010). ApoE polymorphisms and diarrheal outcomes in Brazilian shanty town children. Brazilian J Med Biol Res..

[131] Wozniak MA, Itzhaki RF, Faragher EB, James MW, Ryder SD, Irving WL (2002). Apolipoprotein E-epsilon 4 protects against severe liver disease caused by hepatitis C virus. Hepatology.

[132] Wozniak MA, Faragher EB, Todd JA, Koram KA, Riley EM, Itzhaki RF (2003). Does apolipoprotein E polymorphism influence susceptibility to malaria?. J Med Genet..

[133] Fujioka H, Phelix CF, Friedland RP, Zhu X, Perry EA, Castellani RJ, Perry G (2013). Apolipoprotein E4 prevents growth of malaria at the intraerythrocyte stage: implications for differences in racial susceptibility to Alzheimer’s disease. J Health Care Poor Underserved.

[134] Burt TD, Agan BK, Marconi VC, He W, Kulkarni H, Mold JE, Cavrois M, Huang Y, Mahley RW, Dolan MJ, McCune JM, Ahuja SK (2008). Apolipoprotein (apo) E4 enhances HIV-1 cell entry in vitro, and the APOE ε4/ε4 genotype accelerates HIV disease progression. Proc Natl Acad Sci U S A.

[135] Burgos JS, Ramirez C, Sastre I, Bullido MJ, Valdivieso F (2003). ApoE4 is more efficient than E3 in brain access by herpes simplex virus type 1. Neuroreport.

[136] Kuhlmann I, Minihane AM, Huebbe P, Nebel A, Rimbach G (2010). Apolipoprotein E genotype and hepatitis C, HIV and herpes simplex disease risk: a literature review. Lipids Health Dis..

[137] Mahley RW, Huang Y (2012). Small-molecule structure correctors target abnormal protein structure and function: structure corrector rescue of apolipoprotein E4–associated neuropathology. J Med Chem..

[138] Panza F, Solfrizzi V, Colacicco AM, Basile AM, D’Introno A, Capurso C, Sabba M, Capurso S, Capurso A (2003). Apolipoprotein E (APOE) polymorphism influences serum APOE levels in Alzheimer’s disease patients and centenarians. Neuroreport.

[139] Gupta VB, Laws SM, Villemagne VL, Ames D, Bush AI, Ellis KA, Lui JK, Masters C, Rowe CC, Szoeke C, Taddei K, Martins RN (2011). Plasma apolipoprotein E and Alzheimer disease risk: the AIBL study of aging. Neurology.

[140] Lehtimäki T, Pirttilä T, Mehta PD, Wisniewski HM, Frey H, Nikkari T (1995). Apolipoprotein E (apoE) polymorphism and its influence on ApoE concentrations in the cerebrospinal fluid in Finnish patients with Alzheimer’s disease. Hum Genet..

[141] Riddell DR, Zhou H, Atchison K, Warwick HK, Atkinson PJ, Jefferson J, Xu L, Aschmies S, Kirksey Y, Hu Y, Wagner E, Parratt A, Xu J, Li Z, Zaleska MM, Jacobsen JS, Pangalos MN, Reinhart PH (2008). Impact of apolipoprotein E (ApoE) polymorphism on brain ApoE levels. J Neurosci..

[142] Beffert U, Cohn JS, Petit-Turcotte C, Tremblay M, Aumont N, Ramassamy C, Davignon J, Poirier J (1999). Apolipoprotein E and β-amyloid levels in the hippocampus and frontal cortex of Alzheimer’s disease subjects are disease-related and apolipoprotein E genotype dependent. Brain Res..

[143] Bertrand P, Poirier J, Oda T, Finch CE, Pasinetti GM (1995). Association of apolipoprotein E genotype with brain levels of apolipoprotein E and apolipoprotein J (clusterin) in Alzheimer disease. Brain Res Mol Brain Res..

[144] Glöckner F, Meske V, Ohm TG (2002). Genotype-related differences of hippocampal apolipoprotein E levels only in early stages of neuropathological changes in Alzheimer’s disease. Neuroscience.

[145] Poirier J, Miron J, Picard C, Gormley P, Théroux L, Breitner J, Dea D (2014). Apolipoprotein E and lipid homeostasis in the etiology and treatment of sporadic Alzheimer’s disease. Neurobiol Aging.

[146] Aleong R, Aumont N, Dea D, Poirier J (2003). Non‐steroidal anti‐inflammatory drugs mediate increased in vitro glial expression of apolipoprotein E protein. Eur J Neurosci..

[147] Champagne D, Pearson D, Dea D, Rochford J, Poirier J (2003). The cholesterol-lowering drug probucol increases apolipoprotein E production in the hippocampus of aged rats: implications for Alzheimer’s disease. Neuroscience.

[148] Riddell DR, Zhou H, Comery TA, Kouranova E, Lo CF, Warwick HK, Ring RH, Kirksey Y, Aschmies S, Xu J, Kubek K, Hirst WD, Gonzales C, Chen Y, Murphy E, Leonard S, Vasylyev D, Oganesian A, Martone RL, Pangalos MN, Reinhart PH, Jacobsen JS (2007). The LXR agonist TO901317 selectively lowers hippocampal Abeta42 and improves memory in the Tg2576 mouse model of Alzheimer’s disease. Mol Cell Neurosci..

[149] Cramer PE, Cirrito JR, Wesson DW, Lee CYD, Karlo JC, Zinn AE, Casali BT, Restivo JL, Goebel WD, James MJ, Brunden KR, Wilson DA, Landreth GE (2012). ApoE-directed therapeutics rapidly clear β-amyloid and reverse deficits in AD mouse models. Science.

[150] McAsey ME, Cady C, Jackson LM, Li M, Randall S, Nathan BP, Struble RG (2006). Time course of response to estradiol replacement in ovariectomized mice: brain apolipoprotein E and synaptophysin transiently increase and glial fibrillary acidic protein is suppressed. Exp Neurol..

[151] Tesseur I, De Strooper B (2013). When the dust settles: what did we learn from the bexarotene discussion?. Alzheimers Res Ther..

[152] LaClair KD, Manaye KF, Lee DL, Allard JS, Savonenko AV, Troncoso JC, Wong PC (2013). Treatment with bexarotene, a compound that increases apolipoprotein-E, provides no cognitive benefit in mutant APP/PS1 mice. Mol Neurodegener..

[153] Lynch JR, Tang W, Wang H, Vitek MP, Bennett ER, Sullivan PM, Warner DS, Laskowitz DT (2003). APOE genotype and an ApoE-mimetic peptide modify the systemic and central nervous system inflammatory response. J Biol Chem..

[154] Lynch JR, Wang H, Mace B, Leinenweber S, Warner DS, Bennett ER, Vitek MP, McKenna S, Laskowitz DT (2005). A novel therapeutic derived from apolipoprotein E reduces brain inflammation and improves outcome after closed head injury. Exp Neurol..

[155] Bien-Ly N, Gillespie AK, Walker D, Yoon SY, Huang Y (2012). Reducing human apolipoprotein E levels attenuates age-dependent Aβ accumulation in mutant human amyloid precursor protein transgenic mice. J Neurosci..

[156] Chen HK, Liu Z, Meyer-Franke A, Brodbeck J, Miranda RD, McGuire JG, Pleiss MA, Ji ZS, Balestra ME, Walker DW, Xu Q, Jeong DE, Budamagunta MS, Voss JC, Freedman SB, Weisgraber KH, Huang Y, Mahley RW (2012). Small molecule structure correctors abolish detrimental effects of apolipoprotein E4 in cultured neurons. J Biol Chem..

[157] Ye S, Huang Y, Müllendorff K, Dong L, Giedt G, Meng EC, Cohen FE, Kuntz ID, Weisgraber KH, Mahley RW (2005). Apolipoprotein (apo) E4 enhances amyloid beta peptide production in cultured neuronal cells: apoE structure as a potential therapeutic target. Proc Natl Acad Sci U S A.

[158] Minihane AM, Khan S, Leigh-Firbank EC, Talmud P, Wright JW, Murphy MC, Griffin BA, Williams CM (2000). ApoE polymorphism and fish oil supplementation in subjects with an atherogenic lipoprotein phenotype. Arterioscler Thromb Vasc Biol..

[159] Caslake MJ, Miles EA, Kofler BM, Lietz G, Curtis P, Armah CK, Kimber AC, Grew JP, Farrell L, Stannard J, Napper FL, Sala-Vila A, West AL, Mathers JC, Packard C, Williams CM, Calder PC, Minihane AM (2008). Effect of sex and genotype on cardiovascular biomarker response to fish oils: the FINGEN Study. Am J Clin Nutr..

[160] Egert S, Boesch-Saadatmandi C, Wolffram S, Rimbach G, Müller MJ (2010). Serum lipid and blood pressure responses to quercetin vary in overweight patients by apolipoprotein E genotype. J Nutr..

[161] Boesch-Saadatmandi C, Wolffram S, Minihane AM, Rimbach G (2009). Effect of apoE genotype and dietary quercetin on blood lipids and TNF-α levels in apoE3 and apoE4 targeted gene replacement mice. Br J Nutr..

[162] Huebbe P, Jofre‐Monseny L, Rimbach G (2009). Alpha‐tocopherol transport in the lung is affected by the apoE genotype—studies in transgenic apoE3 and apoE4 mice. IUBMB Life.

[163] Huebbe P, Lodge JK, Rimbach G (2010). Implications of apolipoprotein E genotype on inflammation and vitamin E status. Mol Nutr Food Res..

[164] Egert S, Rimbach G, Huebbe P (2012). ApoE genotype: from geographic distribution to function and responsiveness to dietary factors. Proc Nutr Soc..

[165] Huebbe P, Nebel A, Siegert S, Moehring J, Boesch-Saadatmandi C, Most E, Pallauf J, Egert S, Müller MJ, Schreiber S, Nöthlings U, Rimbach G (2011). APOE ε4 is associated with higher vitamin D levels in targeted replacement mice and humans. FASEB J..

[166] Nichol K, Deeny SP, Seif J, Camaclang K, Cotman CW (2009). Exercise improves cognition and hippocampal plasticity in APOE ε4 mice. Alzheimer’s Dement..

[167] Gustavsson J, Mehlig K, Leander K, Strandhagen E, Björck L, Thelle DS, Lissner L, Blennow K, Zetterberg H, Nyberg F (2012). Interaction of apolipoprotein E genotype with smoking and physical inactivity on coronary heart disease risk in men and women. Atherosclerosis.

[168] Tagalakis AD, Dickson JG, Owen JS, Simons JP (2005). Correction of the neuropathogenic human apolipoprotein E4 (APOE4) gene to APOE3 in vitro using synthetic RNA/DNA oligonucleotides (chimeraplasts). J Mol Neurosci..

[169] Carroll D (2012). A CRISPR approach to gene targeting. Mol Ther..

[170] Huebbe P, Dose J, Schloesser A, Campbell G, Glüer CC, Gupta Y, Ibrahim S, Minihane AM, Baines JF, Nebel A, Rimbach G (2015). Apolipoprotein E (APOE) genotype regulates body weight and fatty acid utilization—studies in gene‐targeted replacement mice. Mol Nutr Food Res..

[171] McKay GJ, Patterson CC, Chakravarthy U, Dasari S, Klaver CC, Vingerling JR, Ho L, de Jong PTVM, Fletcher AE, Young IS, Seland JH, Rahu M, Soubrane G, Tomazzoli L, Topouzis F, Vioque J, Hingorani AD, Sofat R, Dean M, Sawitzke J, Seddon JM, Peter I, Webster AR, Moore AT, Yates JR, Cipriani V, Fritsche LG, Weber BH, Keilhauer CN, Lotery AJ, Ennis S, Klein ML, Francis PJ, Stambolian D, Orlin A, Gorin MB, Weeks DE, Kuo CL, Swaroop A, Othman M, Kanda A, Chen W, Abecasis GR, Wright AF, Hayward C, Baird PN, Guymer RH, Attia J, Thakkinstian A, Silvestri G (2011). Evidence of association of APOE with age‐related macular degeneration: a pooled analysis of 15 studies. Hum Mutat.

[172] Theendakara V, Peters-Libeu CA, Spilman P, Poksay KS, Bredesen DE, Rao RV (2016). Direct transcriptional effects of apolipoprotein E. J Neurosci..

[173] Turchan-Cholewo J, Liu Y, Gartner S, Reid R, Jie C, Peng X, Chen KCC, Chauhan A, Haughey N, Cutler R, Mattson MP, Pardo C, Conant K, Sacktor N, McArthur JC, Hauser KF, Gairola C, Nath A (2006). Increased vulnerability of ApoE4 neurons to HIV proteins and opiates: protection by diosgenin and l-deprenyl. Neurobiol Dis..

[174] Valla J, Yaari R, Wolf AB, Kusne Y, Beach TG, Roher AE, Corneveaux JJ, Huentelman MJ, Caselli RJ, Reiman EM (2010). Reduced posterior cingulate mitochondrial activity in expired young adult carriers of the APOE ε4 allele, the major late-onset Alzheimer’s susceptibility gene. J Alzheimer’s Dis..

[175] Tambini MD, Pera M, Kanter E, Yang H, Guardia‐Laguarta C, Holtzman D, Sulzer D, Area‐Gomez E, Schon EA (2016). ApoE4 upregulates the activity of mitochondria‐associated ER membranes. EMBO Rep..

[176] Mahley RW, Huang Y, Rall SC (1999). Pathogenesis of type III hyperlipoproteinemia (dysbetalipoproteinemia): questions, quandaries, and paradoxes. J Lipid Res..

